# Cuticular Hydrocarbons of Six Geographic Populations of *Ips subelongauts* in Northeastern China: Similarities and Evolutionary Hints

**DOI:** 10.3390/insects16040384

**Published:** 2025-04-03

**Authors:** Yuge Zhao, Chao Wang, Xinmeng Liu, Xu Lin, Dongdong Chu, Junyi Ding, Xiangbo Kong, Dafeng Chen

**Affiliations:** 1 State Forestry and Grassland Administration Key Laboratory of Silviculture in Downstream Areas of the Yellow River, College of Forestry, Shandong Agricultural University, Tai’an 271018, China; zyg15824990632@163.com (Y.Z.); liuxinmeng0125@163.com (X.L.); 17863640509@163.com (X.L.); dingjunyi2119@hotmail.com (J.D.); 2 Key Laboratory of Forest Protection of National Forestry and Grassland Administration, Ecology and Nature Conservation Institute, Chinese Academy of Forestry, Beijing 100091, China; 17837194892@163.com; 3 College of Horticulture Science and Engineering, Shandong Agricultural University, Tai’an 271018, China; 13563838002@163.com

**Keywords:** bark beetles, cuticular hydrocarbon, chemotaxonomic characteristics, population divergence

## Abstract

The Asian larch bark beetle, *I. subelongatus* Motschulsky, is a severe pest of various *Larix* species in its natural range. This study reports the degree of similarity among cuticular hydrocarbon (CHC) profiles of six populations of *I. subelongatus* in northeastern China. Thirty individual or isomeric mixtures of hydrocarbons were identified by solid-phase microextraction (SPME) coupled with gas chromatography–mass spectrometry (GC–MS). The hydrocarbon components consist of straight-chain alkanes, alkenes, and methyl-branched hydrocarbons with carbon chain lengths ranging from 24 to 31. Among these, four CHCs (*n*-C_25_, 9-C_27:1_, *n*-C_27_, and 3-meC_27_) can serve as chemotaxonomic markers to identify *I. subelongatus*. No qualitative differences were detected between males and females in any of the six populations, but significant quantitative differences were observed in some CHCs. Phylogenetic analysis based on CHC profiles showed only minor differences compared to analysis based on partial mtDNA COI sequences regarding bark beetle species affinity. These results establish a rapid chemotaxonomic method and provide a basis for further investigations into the functions of CHCs in *I. subelongatus*.

## Simple Summary

The *Ips* bark beetles are serious wood-boring pests. Accurate and convenient identification of these pests is crucial for forest protection. This study characterizes the cuticular hydrocarbons (CHCs) of *I. subelongatus* in northeastern China, revealing both similarities and differences among populations. The phylogenetic tree constructed using CHCs can distinguish *I. subelongatus* from other bark beetle species, aligning with the nucleic acid-based phylogenetic tree. CHCs are expected to serve as a chemotaxonomic marker for *I. subelongatus*.

## 1. Introduction

Cuticular hydrocarbons (CHCs) are a class of lipids that cover the surface of insect exoskeletons, playing crucial roles in both physiological and ecological functions. They are mainly composed of *n*-alkanes, alkenes, and methyl-branched components, with carbon chain lengths typically ranging from 20 to 40 [1,2,3]. As the first barrier for insects, CHCs can limit water evaporation and prevent pesticides, pathogenic bacteria, and other harmful substances from directly invading the body, thereby greatly enhancing the ability of insects to adapt to harsh environments [4]. More importantly, CHCs play a crucial role in mediating chemical communication signals for various insect behaviors, including species recognition, courtship, mate choice, and maternal care [5,6,7,8]. Meanwhile, they also serve as kairomones, allowing predators to locate and identify prey and thus mediating predator–prey interactions [9,10,11,12].

Correct species delimitation is the basis for studying the biology, physiology, and ecology of insects, as well as their interactions within populations. Until now, morphologies and phylogenetics have been the most commonly employed methods in this field. However, chemical communication signals, which exhibit species-specificity, have increasingly been recognized as important taxonomic traits of insects, such as sex pheromones and CHCs [13,14,15,16,17]. The use of CHCs as taxonomic traits has several significant advantages. For example, they exhibit a high degree of natural variation due to direct selection, aid in the identification of cryptic species, and remain stable over decades [18]. Compared to morphological traits and genetic markers, CHC profiles are expected to reflect recent events of speciation and reproductive isolation more sensitively. Moreover, variations in CHCs can be detected faster and earlier than changes in species morphology or genetics, which typically occur through slow and long-term processes [16]. However, the use of CHCs as taxonomic traits also has notable drawbacks, such as intraspecific variation and technical challenges [17]. For example, gene silencing or downregulation, rather than complete gene loss, may account for the absence of certain classes of CHCs in specific taxa or species within Hymenoptera [19]. This phenomenon can lead to significant divergence between sister species and complicates efforts to accurately predict their phylogenetic history. Additionally, CHC profiles show considerable variation between different subspecies or geographic populations [20,21,22]. Therefore, further research is needed to improve species delimitation and phylogenetic analysis based on CHCs.

The Asian larch bark beetle, *Ips subelongatus* Motschulsky (Coleoptera: Curculionidae: Scolytinae), is widely distributed in northeastern China, particularly in regions such as the Greater Khingan Range, Lesser Khingan Range, Changbai Mountain, and Yanshan Mountain. This pest primarily infests various species of *Larix* (Pinaceae), including *L. gmelinii* Rupr., *L. olgensis* Henry, *L. principis-rupprechtii* Mayr, *L. kaempferi* Carr., and *L. sibirica* Ledeb. Occasionally, it also infests species of *Pinus* and *Picea*, such as *Pinus koraiensis* Sieb. et Zucc [23,24]. Although *I. subelongatus* typically targets dying or recently felled trees, it can also attack and kill relatively healthy trees during outbreaks [24]. Given the risk of accidental introduction via the timber trade and its potential impact on conifers in non-native regions, *I. subelongatus* was listed as a significant pest in the EPPO A2 alert list in 2005. Previous studies indicated that *I. subelongatus* exhibits population divergence in responses to aggregation pheromones due to differences in distribution range and host species [25,26,27]. However, little is known about the relationship between CHC profiles and population divergence in this species. To date, there are no relevant studies on the CHCs of *I. subelongatus*, an important chemical communication signal.

The objectives of this study were to investigate whether there are differences in the CHC profiles among six populations of *I. sublongatus* in northeastern China and to determine whether CHC profiles can be used as a basis for the chemical classification of this species. Specifically, we characterized the representative CHC profiles of different geographic populations using gas chromatography–mass spectrometry (GC–MS). Additionally, we constructed a phylogenetic tree based on CHC profiles and compared it with a tree constructed from partial COI (cytochrome c oxidase subunit I) sequences. Finally, we aimed to develop a convenient and practical method for distinguishing species or identifying new species based on CHC analyses, which may improve the effectiveness of comprehensive prevention and control measures for forest pests.

## 2. Methods and Materials

### 2.1. Sources of I. subelongatus

**Figure 1:**
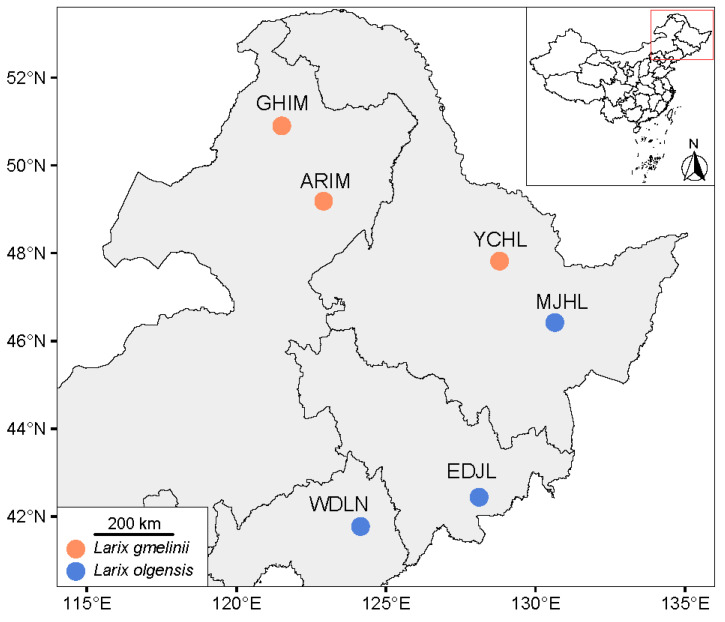
Collection sites for *I. subelongatus* in northeastern China. The map was obtained from Tianditu (https://cloudcenter.tianditu.gov.cn/administrativeDivision/, accessed on 3 February 2025, Figure number GS (2024)0650). Names of localities and acronyms for regions correspond to those in Table S1.

Live specimens of *I. subelongatus* were collected from their host plants and shipped to the laboratory at Shandong Agriculture University, where they were stored at 4 °C in a refrigerator. This temperature was chosen to ensure that the CHC profiles of the live specimens would not be altered. The collection sites and host associations for each population are summarized in Figure 1 and Table S1. Due to limitations in sample availability, only six individuals of *I. subelongatus* were collected from the ARIM area.

### 2.2. Chemicals

The multi-state hydrocarbon window defining the standard including n-alkanes from C_8_ to C_40_ (CDAA-M-690038-HC) was purchased from Anpel Lab-mall Inc. (Shanghai, China). Analytical standards of n-C_20_, n-C_25_, n-C_30_, and n-C_35_ were obtained from Sigma-Aldrich Trading Company (Shanghai, China). The analytical standards 9-C_27:1_, 7-C_27:1_, 9-C_29:1_, and 7-C_29:1_ were generously provided by Dr. Jin Ge (State Key Laboratory of Integrated Management of Pest Insects and Rodents, Institute of Zoology, Chinese Academy of Sciences, Beijing, China). High-performance liquid chromatography (HPLC)-grade dichloromethane and hexane were sourced from Fisher Scientific Company (Geel, Belgium). Methyl disulfide (C_2_H_6_S_2_), iodine (I_2_), sodium thiosulfate (Na_2_S_2_O_3_), and anhydrous sodium sulfate (Na_2_SO_4_) were purchased from Shanghai Macklin Biochemical Co., Ltd. (Shanghai, China).

### 2.3. Sampling of Cuticular Hydrocarbons

Prior to solid-phase microextraction (SPME) of CHCs in the laboratory, live bark beetles were removed from the refrigerator and acclimatized at room temperature for 2 h. A Supelco 70 μm (film thickness) CAR/DVB fiber (lot no. 57336-U; Yellow–Green, Bellefonte, PA, USA) was used to collect CHC samples from individual beetles. We also attempted to collect cuticular hydrocarbons using polydimethylsiloxane (PDMS) fibers. However, the extraction efficiency of PDMS fibers was significantly inferior to that of the polar CAR/DVB fiber. The fiber was activated at 250 °C for 10 min in the inlet of the gas chromatography (GC) before use. Live bark beetles were carefully fixed with gloved fingers, and the fiber was gently rubbed over the dorsal surface of the elytra for 20 s. The SPME fiber was then immediately inserted into the injection port of the GC–MS for chemical analysis. To calculate Kováts retention indices, a 1 μL aliquot of a mixture containing eicosane (*n*-C_20_), pentacosane (*n*-C_25_), triacontane (*n*-C_30_), and pentatriacontane (*n*-C_35_) (5 ng/μL each in dichloromethane) was added to the CAR/DVB fiber and then analyzed by GC–MS. Additionally, a 1 μL aliquot of an *n*-alkane (C_8_-C_40_) standard was directly analyzed by GC–MS.

### 2.4. DMDS Derivatization Reaction

The dimethyl disulfide (DMDS) derivatives were prepared following the procedure reported by Richter et al. [20], with minor modifications. The elytra of bark beetles (20 pieces) were removed and immersed in 200 μL of hexane for 15 min. The sample was then concentrated to 20 μL for the DMDS reaction. To initiate the reaction, 5 μL of an iodine solution (60 mg I_2_ in 1 mL of hexane) and 50 μL of DMDS were added to the sample solution. The reaction was carried out overnight in a 40 °C water bath. Subsequently, 200 μL of hexane and 100 μL of 5% sodium thiosulfate (Na_2_S_2_O_3,_ in deionized water) were added to the reaction mixture. The organic phase was separated, washed twice with deionized water, and dried by adding an appropriate amount of anhydrous sodium sulfate (Na_2_SO_4_) for 30 min. Additionally, DMDS derivatization reactions were performed on the standards 9-C_27:1_, 7-C_27:1_, 9-C_29:1_, and 7-C_29:1_ (Figure S2). Finally, all reaction products were concentrated to 2 μL for GC–MS analysis.

### 2.5. Cuticular Hydrocarbon Analyses with GC–MS

The GC–MS analysis was performed on SPME samples using a Finnigan Trace DSQ GC–MS system equipped with a DB-5MS capillary column (30 m × 0.25 mm i.d. × 0.25 μm film phase; J&W Scientific, Folsom, CA, USA). The compounds were eluted from the column using a programmed temperature gradient, starting at 60 °C (held for 1 min), followed by an increase to 300 °C at a rate of 6 °C/min, and then maintained isothermally at 300 °C for 10 min. To determine the double bond position of the alkenes, the alkene standards and DMDS derivatization products were eluted using an alternative temperature gradient, starting at 60 °C (held for 1 min), increasing to 150 °C at a rate of 10 °C/min, then to 312 °C at a rate of 4 °C/min, and finally held isothermally at 312 °C for 10 min. Mass spectra were acquired by scanning from 41 to 560 amu using electron impact ionization (EI; 70 eV). Helium was used as the carrier gas at a flow rate of 1.0 mL/min. The injector, ion source, and transfer line temperatures were maintained at 220 °C, 250 °C, and 250 °C, respectively. The CAR/DVB fibers were desorbed in the injection port for 1 min in splitless mode. The CHCs were identified by comparing the mass spectra with those in the NIST11 library and by matching the Kováts retention indices (KIs) with the relevant literature data. The KIs were calculated by comparing the retention times of the CHCs in the extracts with those of hydrocarbon standards [21].

### 2.6. Data Analysis

The mean relative amounts of CHCs were calculated using area normalization methods for the peaks [28]. To visualize the distribution of hydrocarbons across the six regions, we plotted bar stacking charts using the percentage of peak area with GraphPad Prism 9 software and generated heatmaps with cluster analysis using PAST 4.16. The mean relative percentage of CHCs between males and females was analyzed using Student’s *t*-test. One-way ANOVA followed by Student–Newman–Keuls’s post hoc test was used for multi-group comparisons across the six regions by SPSS 18.0. To further elucidate differences in CHC profiles among the six regions, non-metric multidimensional scaling (NMDS) analysis based on the Bray–Curtis distance was performed using PAST (Paleontological Statistics, version 4.16) software. Additionally, permutational multivariate analysis of variance (PERMANOVA) was conducted using R 4.4.2 software to evaluate significant differences between regions.

To demonstrate differences among species, principal component analysis (PCA), NMDS, and phylogenetic tree analysis were performed [29]. CHC profiles of other bark beetles were compiled from previous studies [30,31]. For the population phylogenetic analysis, traits included all statistical CHC components coded as not detected (0) or present in the following relative quantities: <0.5% (1); 0.5–1% (2); 1–5% (3); or >5% (4). A data matrix was constructed, where each row represented a species, each column corresponded to a CHC component, and the values were assigned based on their relative percentages. The phylogenetic tree was constructed using the Bray–Curtis distance and the unweighted pair group method with arithmetic mean (UPGMA) in MEGA 7.0. NMDS was also performed on the normalized CHC dataset using the Bray–Curtis dissimilarity distance [32].

Mitochondrial DNA cytochrome oxidase subunit I (mtDNA COI) data were obtained from our previous studies [25]. The sequences were imported into MEGA 7.0 for alignment, with both ends of the sequence matrix aligned. After importing the aligned FASTA file into MEGA, phylogenetic trees were constructed using the maximum likelihood (ML) method. The ML tree was bootstrapped 1000 times to calculate the support rate. To visualize large differences in COI data among species, PCA was performed on the COI dataset using the Bray–Curtis dissimilarity distance.

## 3. Results

### 3.1. Hydrocarbon Identification

Thirty hydrocarbon components, with carbon chain lengths typically ranging from 24 to 31, were identified by GC–MS in the surface lipids of adult *I. subelongauts* from six geographic populations in northeastern China (Figure 1). The hydrocarbons were classified into four major groups: *n*-alkanes (6 components), alkenes (6 components), monomethyl-branched alkanes (13 compounds), and dimethyl-branched alkanes (5 compounds) (Table 1 and Table 2). Internally branched methyl alkanes were identified by analyzing fragmentation patterns, calculating Kováts retention indices, and comparing mass spectra with those of the authentic standards (Figure S1). Alkenes were identified by analyzing the ionic fragments of DMDS derivatization reaction products.

**Table 1:** Identification and percentage composition of cuticular hydrocarbons from elytra surface lipids between the sexes of *I. subelongatus* analyzed by SPME-GC–MS.

GC Peak No.	t_R_/min	KIs	Cuticular Hydrocarbon	Diagnostic EI Ions	% Total Hydrocarbons (Mean ± SE)	
					Male (*N* = 30)	Female (*N* = 28)
1	34.04	2480	#7-pentacosene (7-C_25:1_)	83, 97, 111, 350	0.6 ± 0.1	0.6 ± 0.1
2	34.29	2500	※pentacosane (*n*-C_25_)	352	17.5 ± 1.6 **	11.1 ± 1.4
3	35.21	2572	3-methyl-pentacosane (3-meC_25_)	337, 366	1.4 ± 0.1	1.3 ± 0.1
4	35.56	2600	hexacosane (*n*-C_26_)	366	3.1 ± 0.4	2.7 ± 0.4
5	36.27	2657	4-methyl-hexacosane (4-meC_26_)	43, 71, 337, 380	0.4 ± 0.0	0.6 ± 0.1
6	36.52	2677	#9-heptacosene (9-C_27:1_)	83, 97, 111, 378	16.8 ± 1.7	15.8 ± 2.0
7	36.6	2684	#7-heptacosene (7-C_27:1_)	83, 97, 111, 378	5.3 ± 0.4	4.8 ± 0.6
8	36.8	2700	※heptacosane (*n*-C_27_)	380	21.8 ± 1.6	19.5 ± 1.7
9	37.19	2733	11-methyl-heptacosane (11-meC_27_)	168, 252	0.7 ± 0.1	0.9 ± 0.1
10	37.29	2741	7-methyl-heptacosane (7-meC_27_)	112, 309	0.2 ± 0.0 **	0.4 ± 0.0
11	37.39	2750	5-methyl-heptacosane (5-meC_27_)	85, 337	2.0 ± 0.1	2.4 ± 0.2
12	37.68	2774	3-methyl-heptacosane (3-meC_27_)	365, 394	7.0 ± 0.5	7.9 ± 0.5
13	37.99	2800	octacosane (*n*-C28)	394	1.7 ± 0.2	2.0 ± 0.2
14	38.09	2809	3,7-dimethyl-heptacosane (3,7-dimeC_27_)	393, 379, 126, 308	1.6 ± 0.1	1.7 ± 0.1
15	38.67	2858	4-methyl-octacosane (4-meC_28_)	43, 71, 365	1.1 ± 0.1 *	1.7 ± 0.2
16	38.92	2879	#9-nonacosene (9-C_29:1_)	406, 83, 97, 111	2.0 ± 0.2	2.3 ± 0.2
17	38.99	2885	#7-nonacosene (7-C_29:1_)	406, 71, 83, 97, 111	1.4 ± 0.1	1.6 ± 0.1
18	39.16	2900	※nonacosane (*n*-C_29_)	408	2.4 ± 0.1	2.8 ± 0.2
19	39.5	2930	13-methyl-nonacosane (13-meC_29_)	196, 224, 252, 407	1.1 ± 0.3	1.6 ± 0.4
20	39.6	2938	7-methyl-nonacosane (7-meC_29_)	112, 337, 407	1.2 ± 0.2 *	2.0 ± 0.3
21	39.71	2948	5-methyl-nonacosane (5-meC_29_)	85, 365, 407	1.2 ± 0.2	1.6 ± 0.2
22	39.83	2958	9,13-dimethyl-nonacosane (9,13-dimeC_29_)	421, 323, 252, 211, 140	0.8 ± 0.2	1.1 ± 0.3
23	40.04	2977	5,X-dimethyl-nonacosane (5,X-dimeC_29_)	85, 196, 211, 379, 421	2.5 ± 0.7	4.2 ± 0.9
24	40.31	3000	※triacontane (*n*-C_30_)	422	0.5 ± 0.1	0.5 ± 0.1
25	41.15	3073	7-hentriacontene (7-C_31:1_)	83, 97, 111, 434	0.5 ± 0.1	0.5 ± 0.1
26	41.71	3121	15-methyl-hentriacontane (15-meC_31_)	196, 224, 252	1.6 ± 0.5	2.6 ± 0.6
27	41.84	3133	7-methyl-hentriacontane (7-meC_31_)	112, 365, 436	0.4 ± 0.1	0.7 ± 0.2
28	42.04	3150	11,15-dimethyl-hentriacontane (11,15-meC_31_)	168, 239, 252, 323	0.7 ± 0.2	1.1 ± 0.3
29	42.19	3163	4-methyl-hentriacontane (4-meC_31_)	71, 379, 450	0.9 ± 0.2	1.5 ± 0.4
30	42.3	3173	5,17-dimethyl-hentriacontane (5,17-dimeC_31_)	85, 126, 168, 225, 267, 407	1.7 ± 0.5	2.7 ± 0.7

Note: # indicates that the double bond position was identified by the DMDS derivatives. ※ indicates that the hydrocarbon was identified by authentic standards. Retention times and peak numbers correspond to those indicated in Figure 2. Abbreviations for the compounds are shown in parentheses. KIs refers to Kováts indices. Significant differences are based on Student’s *t*-test; * *p* < 0.05; ** *p* < 0.01.

**Table 2:** Identification and percentage composition of cuticular hydrocarbons from the elytra surface lipids of *I. subelongatus* across six geographical populations.

GC Peak No.	CHCs		% Total Hydrocarbons (Mean ± SE)				
		MJHL *N* = 10	WDLN *N* = 12	GHIM *N* = 10	YCHL *N* = 8	EDJL *N* = 12	ARIM *N* = 6
1	#7-C_25:1_	0.7 ± 0.1 a	0.9 ± 0.1 a	0.1 ± 0.1 b	0.1 ± 0.0 b	0.7 ± 0.1 a	0.8 ± 0.1 a
2	※*n*-C_25_	12.3 ± 1.3 b	17.1 ± 1.9 b	6.5 ± 0.9 c	5.2 ± 0.8 c	26.8 ± 1.3 a	13.4 ± 0.8 b
3	3-meC_25_	1.7 ± 0.1 a	1.6 ± 0.1 a	0.8 ± 0.1 b	0.7 ± 0.1 b	1.7 ± 0.1 a	1.5 ± 0.2 ab
4	*n*-C_26_	5.6 ± 0.5 a	4.2 ± 0.4 a	1.2 ± 0.2 c	0.9 ± 0.1 c	2.6 ± 0.1 b	1.6 ± 0.3 bc
5	4-meC_26_	0.7 ± 0.1 a	0.5 ± 0.1 a	0.4 ± 0.1 ab	0.8 ± 0.5 ab	0.3 ± 0.1 b	0.5 ± 0.1 ab
6	#9-C_27:1_	13.5 ± 0.8 c	18.6 ± 1.4 bc	10.1 ± 1.4 c	4.4 ± 0.4 d	17.6 ± 0.7 b	39.4 ± 2.2 a
7	#7-C_27:1_	5.3 ± 0.5 a	7.4 ± 0.7 a	2.4 ± 0.3 b	1.8 ± 0.2 b	6.1 ± 0.5 a	6.7 ± 0.6 a
8	※*n*-C_27_	23.5 ± 1.8 b	27.6 ± 1.2 a	14.1 ± 1.4 c	8.1 ± 1.1 d	29.3 ± 1.0 a	12.0 ± 0.9 cd
9	11-meC_27_	1.2 ± 0.2 ab	0.3 ± 0.1 bc	1.5 ± 0.1 a	1.7 ± 0.2 a	0.02 ± 0.0 c	0.5 ± 0.1 b
10	7-meC_27_	0.3 ± 0.1 b	0.1 ± 0.1 c	0.5 ± 0.1 ab	0.5 ± 0.1 a	0.1 ± 0.1 c	0.3 ± 0.1 b
11	5-meC_27_	2.9 ± 0.2 a	1.8 ± 0.2 bc	2.7 ± 0.2 a	2.7 ± 0.3 a	1.3 ± 0.1 c	1.9 ± 0.2 b
12	3-meC_27_	11.2 ± 0.3 a	7.3 ± 0.4 bc	8.5 ± 0.5 b	7.0 ± 0.8 bc	4.7 ± 0.4 c	5.0 ± 0.6 c
13	*n*-C_28_	3.3 ± 0.3 a	2.2 ± 0.2 b	1.5 ± 0.2 bc	2.2 ± 0.5 abcd	0.9 ± 0.1 c	0.5 ± 0.1 d
14	3,7-dimeC_27_	2.2 ± 0.2 a	1.2 ± 0.1 b	2.2 ± 0.3 a	1.9 ± 0.1 ab	1.3 ± 0.1 ab	1.6 ± 0.2 ab
15	4-meC_28_	1.7 ± 0.1 b	1.0 ± 0.2 c	1.8 ± 0.1 b	3.2 ± 0.3 a	0.2 ± 0.1 d	1.1 ± 0.1 c
16	#9-C_29:1_	1.5 ± 0.2 d	2.0 ± 0.2 c	2.8 ± 0.2 b	2.2 ± 0.2 bc	1.7 ± 0.1 bcd	3.5 ± 0.3 a
17	#7-C_29:1_	1.2 ± 0.1 bc	1.3 ± 0.1 b	2.1 ± 0.2 a	2.3 ± 0.1 a	0.8 ± 0.1 c	1.8 ± 0.3 a
18	※*n*-C_29_	3.2 ± 0.3 a	3.1 ± 0.3 a	2.4 ± 0.2 a	2.4 ± 0.2 a	2.7 ± 0.2 a	1.0 ± 0.1 b
19	13-meC_29_	0.6 ± 0.2 b	*nd*	3.4 ± 0.3 a	4.3 ± 0.3 a	*nd*	0.6 ± 0.1 b
20	7-meC_29_	1.4 ± 0.2 c	0.6 ± 0.4 c	2.9 ± 0.3 b	4.2 ± 0.2 a	0.1 ± 0.1 d	1.0 ± 0.1 c
21	5-meC_29_	1.7 ± 0.1 c	0.8 ± 0.2 d	2.2 ± 0.2 b	3.1 ± 0.2 a	0.2 ± 0.1 e	0.6 ± 0.1 de
22	9,13-dimeC_29_	*nd*	*nd*	2.5 ± 0.3 a	3.4 ± 0.4 a	*nd*	0.2 ± 0.1 b
23	5,X-dimeC_29_	1.5 ± 0.4 b	0.3 ± 0.1 c	8.2 ± 1.0 a	10.6 ± 0.3 a	0.1 ± 0.1 c	1.4 ± 0.2 b
24	※*n*-C_30_	0.8 ± 0.1 a	*nd*	1.2 ± 0.2 a	0.8 ± 0.1 a	*nd*	0.4 ± 0.1 b
25	9-C_31:1_	0.3 ± 0.1 b	0.1 ± 0.1 b	0.9 ± 0.1 a	1.3 ± 0.19 a	0.2 ± 0.1 b	0.5 ± 0.2 ab
26	15-meC_31_	0.7 ± 0.1 b	*nd*	4.3 ± 0.4 a	8.0 ± 1.08 a	0.3 ± 0.1 b	0.7 ± 0.1 b
27	7-meC_31_	*nd*	*nd*	1.0 ± 0.1 a	2.1 ± 0.14 a	0.2 ± 0.1 b	0.1 ± 0.1 b
28	11,15-dimeC_31_	*nd*	*nd*	2.4 ± 0.3 a	3.4 ± 0.36 a	*nd*	0.2 ± 0.0 b
29	4-meC_31_	0.6 ± 0.1 b	*nd*	3.5 ± 0.5 a	3.2 ± 0.20 a	*nd*	0.5 ± 0.1 b
30	5,17-dimeC_31_	0.5 ± 0.1 b	*nd*	6.0 ± 0.6 a	7.2 ± 0.3 a	*nd*	0.7 ± 0.1 b

Note: # indicates that the double bond position was identified by DMDS derivatives. ※ indicates that the hydrocarbon was identified by authentic standards. Retention times and peak numbers are as indicated in Figure 2. The abbreviations for the compounds are shown in parentheses. KIs, Kováts indices; *nd*, not detected. Significant differences were determined using the Student–Newman–Keuls test. Different letters denote significant differences at *p* < 0.05.

**Figure 2:**
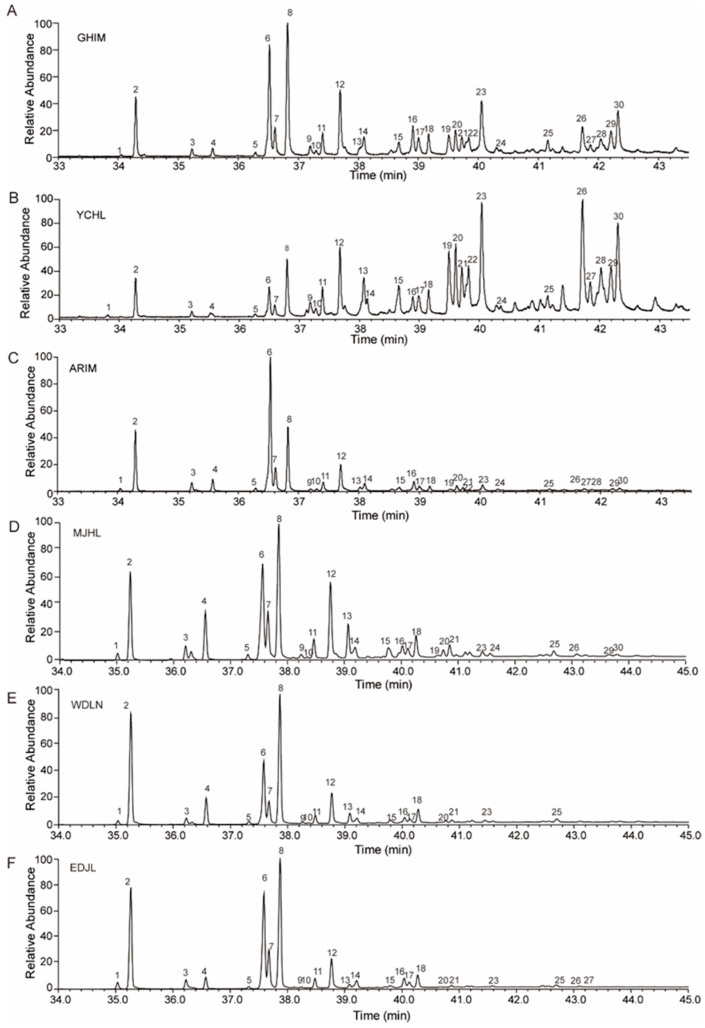
Total ion chromatograms (TICs) of cuticular hydrocarbons from different geographic populations of *I. subelongatus*. (**A**) GHIM, (**B**) YCHL, (**C**) ARIM, (**D**) MJHL, (**E**) WDLN, and (**F**) EDJL. The numbered peaks in each chromatogram correspond to the hydrocarbon components listed in Table 1 and Table 2.

**Figure 3:**
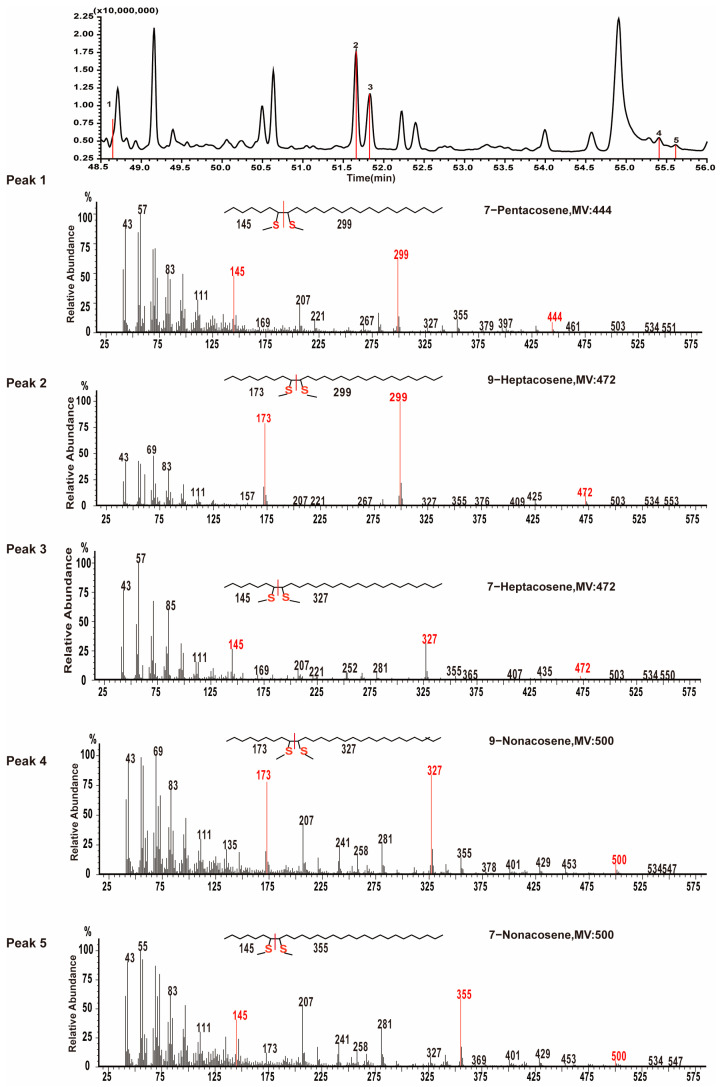
The identification of double-bond positions in pentacosene, heptacosene, and nonacosene. Gas chromatograms show the distribution of DMDS derivatives of 7-pentacosene (Peak 1), 9-heptacosene (Peak 2), 7-heptacosene (Peak 3), 9-nonacosene (Peak 4), and 7-nonacosene (Peak 5). The mass spectra of mono-DMDS adducts for these compounds were provided, with ion fragments related to double-bond positions marked in red.

Specifically, mass spectra containing fragmentation ions at *m*/*z* 145, 299, and 444 (Figure 3, Peak 1) were attributed to thiomethylether addition to carbons 7 and 8 of 7-pentacosene. Fragmentation ions at *m*/*z* 173, 299, and 472 (Figure 3, Peak 2) were identified as resulting from thiomethylether addition to carbons 9 and 10 of 9-heptacosene. Additionally, fragmentation ions at *m*/*z* 145, 327, and 472 (Figure 3, Peak 3) were assigned to thiomethylether addition to carbons 7 and 8 of 7-heptacosene. Notably, 9-heptacosene eluted earlier than 7-heptacosene. Other identified alkenes included 9-nonacosene (fragmentation ions at *m*/*z* 173, 327, and 500 in Figure 3, Peak 4) and 7-nonacosene (fragmentation ions at *m*/*z* 145, 355, and 500 in Figure 3, Peak 5). CHC extracted samples were analyzed using alkene standards as internal standards, and the addition of internal standards significantly increased the percentages of 9-heptacosene, 7-heptacosene, 9-nonacosene, and 7-nonacosene (Figure S3).

### 3.2. Percentage Composition of Cuticular Hydrocarbons in I. subelongatus

The CHCs primarily consisted of *n*-alkanes (42.3 ± 0.1%), alkenes (25.8 ± 0.1%), monomethyl-branched alkanes (22.3 ± 0.0%), and dimethyl-branched alkanes (9.2 ± 0.0%). Among the identified compounds, pentacosane (*n*-C_25_), 9-heptacosene (9-C_27:1_), heptacosane (*n*-C_27_), and 3-methyl-heptacosane (3-meC_27_) were the most abundant, each comprising more than 7% of the total hydrocarbon profile (Table 1). While most peaks showed no differences in relative abundances between males and females, notable sex-specific variations were observed. Specifically, the percentage of *n*-C25 was significantly higher in males than in females (Table 1). Conversely, the percentage compositions of 7-methyl-heptacosane (7-meC_27_), 4-methyl-octacosane (4-meC_28_), and 7-methyl-nonacosane (7-meC_29_) were significantly higher in females than in males (Table 1). In conclusion, the CHC profiles of *I. subelongatus* exhibited no significant qualitative differences between males and females. However, quantitative differences in specific hydrocarbon components suggest sex-specific variations in the abundance of certain CHCs.

**Figure 4:**
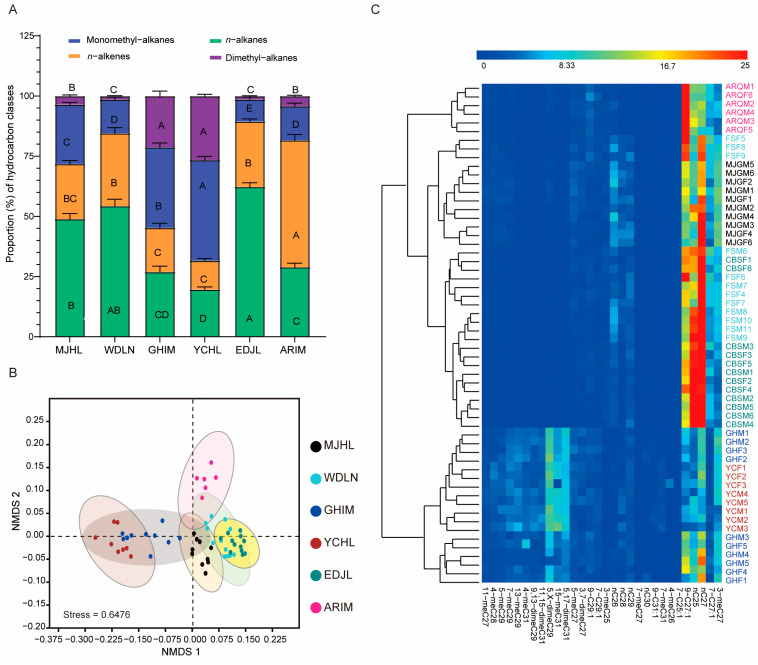
Analysis of CHC variations of *I. subelongatus* among different geographic populations. (**A**) The mean percentage (±SE) of four types of cuticular hydrocarbon composition from different geographic populations. Bars labeled with different capital letters indicate significant differences (*p* < 0.05) among populations, as determined by the Student–Newman–Keuls test. (**B**) Non-metric multidimensional scaling analysis (NMDS) based on Bray–Curtis dissimilarity in cuticular hydrocarbons. (**C**) Heatmap of the percentage composition of cuticular hydrocarbons from individuals of different populations, accompanied by cluster analysis.

The composition of *n*-alkanes, alkenes, monomethyl-branched alkanes, and dimethyl-branched alkanes exhibited clear differences among the various populations (Figure 4A). For example, the percentage composition of *n*-alkanes in the MJHL, WDLN, and EDJL populations was approximately 50%, while it was only about 20% in the GHIM, YCHL, and ARIM populations. The percentage composition of alkenes in the ARIM population reached 52.7%, which was significantly higher than in the other populations. Additionally, the percentage composition of both monomethyl-branched and dimethyl-branched alkanes was significantly higher in the GHIM and YCHL populations compared to the other populations. Overall, the CHC profiles revealed significant differences between the populations, as confirmed by PERMANOVA analysis (R^2^ = 0.61; *p* = 0.001, Table S2).

Some trace CHC components were undetectable in certain populations, while specific hydrocarbon components exhibited significant quantitative differences across different geographic populations (Table 2). For example, six CHC components—13-methyl-nonacosane (13-meC_29_), 9,13-dimethyl-nonacosane (9,13-dimeC_29_), triacontane (*n*-C_30_), 11,15-dimethyl-hentriacontane (11,15-meC_31_), 7-methyl-hentriacontane (7-meC_31_), 4-methyl-hentriacontane (4-meC_31_), and 5,17-dimethyl-hentriacontane (5,17-meC_31_)—were not detected in the WDLN and EDJL populations. Additionally, two of these components (9,13-dimeC_29_ and 4-meC_31_) were also undetectable in the MJHL population (Table 2, Figure 2). However, the percentage composition of 5,17-meC_31_ was approximately 6.0% in the GHIM population and 7.2% in the YCHL populations (Table 2, Figure 2). In contrast, the percentage composition of *n*-C_25_, 9-C_27:1_, *n*-C_27_, and 3-meC_27_ was significantly lower in the GHIM and YCHL populations compared to other populations. The percentage composition of 5,X-dimeC_29_, 15-meC_31_, and 5,17-dimeC_31_ was significantly higher in these populations (Figure 4C). These findings indicate that the CHC profiles of *I. subelongatus* exhibit considerable variation among different geographic populations.

### 3.3. Population Differences in Cuticular Hydrocarbon Profiles

To explore the extensive differences in CHC profiles among geographic populations, we conducted NMDS and cluster analysis based on the percentage composition of the CHC dataset. Both analyses revealed overlap among some geographic populations and between sexes (Stress = 0.6476; Figure 4B,C). The populations were broadly classified into two distinct CHC categories: one comprising the GHIM and YCHL populations, and the other comprising the MJHL, ARIM, WDLN, and EDJL populations (Figure 4C). These two groups exhibited significant differences in their CHC profiles. However, all six populations belong to the same species. Additionally, the ARIM population was clearly separated from the YCHL and EDJL populations, while the YCHL population was well-differentiated from the other four populations, except for GHIM, according to both NMDS and cluster analysis (Figure 4B,C). No distinct separation was observed between males and females (Figure 4C). These results suggest that CHC profiles diverge significantly among geographic populations, with this divergence being considerably greater than the differences observed between sexes.

### 3.4. Cuticular Hydrocarbons as Taxonomic Traits

**Figure 5:**
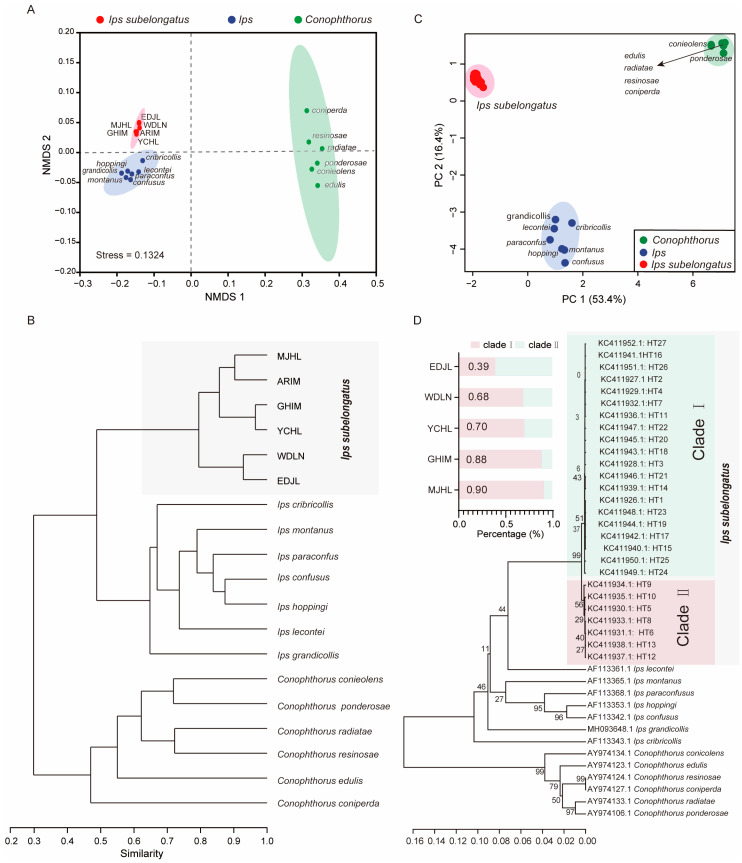
Separation among *I. subelongauts*, *Conophthorus*, and *Ips* species. (**A**) NMDS and (**B**) phylogenetic analyses were conducted based on the CHC profiles of different bark beetle species, including *I*. *subelongatus*, other *Ips* species, and *Conophthorus* species. (**C**) PCA and (**D**) phylogenetic analyses were performed based on the COI gene sequences of different bark beetle species. The bar graph adjacent to the phylogenetic tree illustrates the distribution of COI gene haplotypes among the populations.

To evaluate the potential of CHC profiles as taxonomic traits, NMDS and phylogenetic analyses were performed on the normalized CHC dataset of *I. subelongatus* and other related bark beetles, including species from the genera *Ips* and *Conophthorus*. The results indicated that *I. subelongatus* was distinctly separated from other *Ips* and *Conophthorus* bark beetles, showing a closer relationship to *Ips* than to *Conophthorus* (Stress = 0.1324; Figure 5A,B). Although *Conophthorus* and *Ips* bark beetles were well separated in the analysis, the similarity of CHC profiles was found to be greater within *Ips* species than within *Conophthorus* species (Figure 5A). This suggests that CHC profiles can serve as effective taxonomic traits for distinguishing between closely related bark beetle species, with potential applications in species identification and phylogenetic studies.

PCA and phylogenetic analyses were conducted to assess the consistency between the phylogenetic relationships inferred from CHCs and those based on COI sequences. The first two principal components (PC1 and PC2) accounted for 53.4% and 16.4% of the total variation, respectively, based on the COI sequence (Figure 5C). The topological structures derived from CHCs and COI sequences were broadly similar (Figure 5B,D). However, there were notable differences in the clustering patterns of certain species. *I. subelongatus* was clearly separated from other *Ips* and *Conophthorus* bark beetles along PC1 and PC2. However, some overlap was observed between *Ips* bark beetles, including *I. montanus* and *I. hoppingi*, and *Conophthorus* bark beetles, such as *C. edulis*, *C. radiatae*, *C. resinosae* and, *C. coniperda*, along these PC1 and PC2 (Figure 5C). All mtDNA COI haplotypes of *I. subelongatus* were clustered into two distinct clades (Figure 5D). The distribution ratio of haplotypes between clade I and clade II varied significantly among different populations, with inverse patterns observed in some cases: 4:6 in the EDJL population, 7:3 in the WDLN and YCHL populations, and 9:1 in the GHIM and MJHL populations (Figure 5D).

Additionally, *I. lecontei* and *I. subelongatus* were clustered into a single branch based on the COI sequence analysis. While the overall topological structures based on CHCs and COI sequences showed similarities between the two genera, they also revealed differences among species within the same genus for both *Ips* and *Conophthorus* bark beetles (Figure 5B,D). For example, *C. resinosae* and *C. coniperda* were clustered together based on COI sequences, whereas *C. resinosae* and *C. radiatae* were clustered together based on CHCs (Figure 5B,D). These results indicate that CHCs can serve as taxonomic characters, but the phylogenetic relationships inferred from COI sequences may vary among different bark beetle species.

## 4. Discussion and Conclusions

Our study clearly demonstrated that CHCs vary qualitatively among different geographic populations of *I. subelongatus* in northeastern China. The significant differences in CHC profiles among bark beetle species suggest that these profiles are useful for species classification and can explain geographic variation among populations. Phylogenetic analysis based on CHCs, both interspecific and intraspecific, is generally consistent with that based on mtDNA COI sequences, although there are some minor differences.

CHCs have been established as taxonomic traits in many insect species, including bark beetles [13,30,31], thrips [33], the African cryptic species complex [34], calliphorids [35], ants [36], and termites [37]. CHCs with carbon chains ranging from 25 to 30 have been consistently identified in both *Conophthorus* and *Ips* bark beetles [31,32]. However, *Ips* bark beetles tend to have more overlapping CHCs within the genus compared to *Conophthorus* bark beetles. The CHC profiles of *I. subelongatus* differ markedly from those of other *Ips* bark beetles in both qualitative and quantitative aspects. For example, the relative quantities of 9-C_27:1_ and 3-meC_27_ are above 5% in *I. subelongatus*, but below 5% in other *Ips* bark beetles. Additionally, 7-C_27:1_ and 4-meC_31_ are unique to *I. subelongatus* (Table 1), whereas heptacosadiene, nonacosadiene, 3-meC_26_, 3-meC_28_, 3-meC_29_, and 3-meC_31_ are also found in other *Ips* bark beetles [31]. These results indicate that CHCs differ significantly among species not only in composition but also in proportion. However, the use of CHC profiles as a taxonomic tool has certain limitations due to variations depending on sex, age, environment, diet, and sexual maturity [19,38,39]. In our study, we selected newly emerged overwintered adults to minimize the effects of physiological condition, nutrition, and sexual maturity. Therefore, it is crucial to consider the condition of the insects when assessing differences in the quality and quantity of CHCs between species. Despite these limitations, CHC profiles remain a potentially powerful tool for insect chemotaxonomy across different insect taxa.

In addition to species-specific traits, geographically separated populations may also exhibit qualitative and quantitative differences in CHC profiles, which are influenced by both genetic factors and environmental conditions [40,41]. For example, in *Calliphora vicina*, geographic variation in adult CHC profiles was observed among three populations, with greater climatic differences correlating with increased CHC variation [42]. Similarly, in *Drosophila montana,* differences in CHC profiles were found to be more pronounced between populations than between sexes [43,44]. Our results also confirm this phenomenon, revealing significant qualitative and quantitative differences in CHC profiles among geographic populations of *I. subelongatus*. For example, several hydrocarbons with carbon chain lengths of 29 to 31 were undetectable in the WDLN and EDJL populations but exhibited significantly higher relative abundance in the YCHL and GHIM populations (Figure 2, Table 2). Specifically, high-content hydrocarbons such as *n*-C_25_, 9-C_27:1_, *n*-C_27_, and 3-meC_27_ showed marked differences between geographic populations. For example, the proportion of *n*-C25 was 26.8% in the EDJL population, compared to approximately 5% in the YCHL and GHIM populations. Similarly, the proportion of 9-C_27:1_ was 39.4% in the ARIM population but less than 5% in the YCHL population. Additionally, the proportion of 3-meC_27_ was 11.5% in the MJHL population, while it was less than 5% in the EDJL and ARIM populations.

Although the YCHL, GHIM, and ARIM populations share the same host plants, the YCHL and GHIM populations exhibited greater similarities in CHC profiles than the ARIM population. Conversely, the WDLN, MJHL, and EDJL populations, which also share the same host, showed greater similarity in CHC profiles due to their closer geographical proximity. Indeed, different hosts can lead to distinct CHC profiles of *I. subelongatus*. For example, in *Polistes biglumis*, variations in CHC profiles among different populations followed a pattern consistent with the isolation-by-distance hypothesis [45]. However, our results suggest that multiple factors contribute to the variation in CHC profiles among different populations, including host plant, climate, and environmental conditions. The relative contributions of these factors require further investigation and validation. These qualitative differences among different geographic populations could easily lead to misidentification if relying solely on CHCs as taxonomic traits. CHC profiles exhibit phenotypic plasticity, with both genetic and non-genetic factors contributing to their diversity [46]. Moreover, CHC profiles demonstrate remarkable adaptability to a wide range of biotic and abiotic conditions [47]. Therefore, when using CHCs as taxonomic traits, it is essential to fully account for both qualitative and quantitative differences and emphasize the proportional characteristics of the most important CHCs as fingerprints.

In the present study, cluster analysis based on CHCs revealed that different species within the same genus and different genera within the same family were clustered together. Phylogenetic analyses based on hydrocarbon phenotypes were largely consistent with published phylogenies reconstructed from proteins and nucleic acids, as well as with the morphologically based system of relatedness [48,49]. For example, in ants, CHC traits at the genus, clade, and species group levels were found to be evolutionarily labile and have evolved independently of phylogenetic constraints [50]. In contrast, in *Reticulitermes*, phylogenetic analyses based on mtDNA sequences and CHCs produced trees with very similar topologies [51]. Biogeographic differences in CHC diversity are largely consistent with biochemical variation resulting from colonization history, while post-colonization processes such as drift or selection have also played important roles in the evolution of CHC diversity [52]. To date, phylogenetic assessments based on CHCs have been conducted in numerous insect species, with results often aligning well with established taxonomic relationships. However, in some species, dendrograms based on hydrocarbon data did not perfectly match established taxonomic relationships [53,54,55]. This suggests that the use of CHCs to study phylogeny is species-specific.

Geographic variation in insects is evident in many aspects, especially in morphological, behavioral, genetic, and ecological traits. Currently, phylogenetic analysis based on mitochondrial DNA is the predominant method for assessing geographic variation. Additionally, some studies have used CHCs to investigate the phylogeny of geographic populations. Indeed, a correlation between genetic variation and chemical variation has been established in certain species [56,57,58,59]. However, phylogenetic trees constructed from genetic data do not match those based on CHCs, suggesting that genetic variation does not fully represent the variation observed in CHCs [45,49]. In our study, cluster analysis revealed a high similarity between genetic data and CHCs in *I. subelongatus*. Populations with greater similarity in CHCs also exhibited more comparable haplotypes, despite minor differences. For example, although MJHL and YCHL are geographically closer to each other, the CHCs of YCHL and GHIM are more similar, and their haplotype diversity is more comparable (Figure 5). This slight discrepancy can be easily explained by variations in CHC profiles influenced by different microenvironmental factors. Moreover, the population variation in CHCs shows a remarkable similarity with the divergence in aggregation pheromone responses of *I. subelongatus* [25]. Overall, we suggest that CHC profiles can serve as complementary information to assess genetic structure and geographic variation at the intraspecific level in *I. subelongatus*.

CHCs exhibit sexual dimorphism in quality or quantity in some species and are involved in their mating behavior [60], especially alkenes and methyl-branched alkanes, which play an important role in sex communication [6,61]. Closely related species show similarities in the selection and use of alkenes or methyl-branched alkanes as pheromones, with the carbon chain length, position or number of double bonds or methyl branches varying between species. For example, in the genus *Drosophila*, mono- or dienes with odd carbon chain lengths between 23 and 29 are typically used as pheromones. Male CHC profiles are extremely similar, while female CHC profiles show greater variability between species [41]. In our study, the relative content of *n*-C_25_ was significantly higher in males, while 7-meC_27_, 4-meC_28_, and 7-meC_29_ were significantly higher in females (Table 1). Further studies are needed to verify the possible functions of these five CHCs in sex communication.

In summary, we characterized the CHC profiles of *I. subelongatus* for the first time and observed significant geographic variation of CHCs in northeastern China. This provides a potential method for species identification and phylogenetic analysis based on CHC profiles in bark beetles. Our results offer new insights into the intraspecific and interspecific variation and phylogenetics of bark beetles.

## Data Availability

The original contributions presented in the study are included in the article/Supplementary Materials, further inquiries can be directed to the corresponding authors.

## Supplementary Materials

The following supporting information can be downloaded at: https://www.mdpi.com/article/10.3390/insects16040384/s1, Table S1: Collection data for *I. subelongatus* from northeastern China; Table S2: Pairwise permutational multivariate analysis of variance (PERMANOVA) comparisons of CHCs profiles of *I. subelongatus* from different geographical population; Table S3: The COI haplotypes of *I. subelongatus*. Figure S1: Mass spectra of main CHCs and their representative diagnostic ions and relative abundances; Figure S2: GC-MS showing the distribution of mono-DMDS adducts of standards; Figure S3: Total ion chromatograms (TICs) of sample and samples with heptacosene and nonacosene standards.
