# Supplementary material for: Cuticular Hydrocarbons of Six Geographic Populations of Ips subelongauts in Northeastern China: Similarities and Evolutionary Hints
Source: Insects. 2025 Apr 3;16(4):384. doi: 10.3390/insects16040384 (PMC12027504; doi:10.3390/insects16040384)
Supplement: Supplementary file 1 [file insects-16-00384-s001.zip › insects-3553086-supplementary.pdf]

Table S1. Collection data for *I. subelongatus* from northeastern China.

Population symbol	Locality	Latitude, longitude, and altitude	Collection date	Host plant	Number	
					Male	Female
GHIM	Inner Mongolia: Ecological Research Station, Genhe city	N: 50°54′, E: 121°31′; H: 870 m	2023.7		5	5
ARIM	Inner Mongolia: Dashini Forest Farm, Arongqi city	N: 48°71′, E: 122°55′; H: 501 m	2023.7	*Larix gmelinii*	4	2
YCHL	Heilongjiang province: Youhao Forest Farm, Yichun city	N: 47°49′, E: 128°48′; H: 319 m	2023.7		3	5
MJHL	Heilongjiang province: Mengjiagang Forest Farm, Jiamusi city	N: 46°25′, E: 130°39′; H: 185 m	2023.7		6	4
EDJL	Jilin province: Erdaobaihe town, Antu county	N: 42°26′, E: 128°7′; H: 695m	2023.7	*Larix olgensis*	6	6
WDLN	Liaoning province: Wendaoforest Farm, Funshun city	N: 41°46′, E: 124°9′; H: 209 m	2023.7		6	6

Table S2. Pairwise permutational multivariate analysis of variance (PERMANOVA) comparisons of CHCs profiles of *I. subelongatus* from different geographical population.

Population symbol	GHIM	YCHL	ARIM	MJHL	WDLN
YCHL	0.005				
ARIM	0.001	0.001			
MJHL	0.001	0.001	0.001		
WDLN	0.001	0.001	0.001	0.015	
EDJL	0.001	0.001	0.001	0.001	0.010
adonis $R^2 = 0.61$; P-value = 0.001					

Table S3. The COI haplotypes of *I. subelongatus.*

Population symbol	Collection date	N	Haplotype(individuals)
GHIM	July 2011 and 2012	17	HT2(15), HT6(2)
ARIM	-	-	-
YCHL	August 2011	23	HT2(15), HT6(6), HT8, HT11
MJHL	July 2012	20	HT1(2), HT2(11), HT3, HT6, HT11, HT12, HT16, HT17, HT18
EDJL	July 2012	18	HT1(4), HT5(10), HT6, HT19(2), HT27
WDLN	August 2011	19	HT1(4), HT2(6), HT5(5), HT6, HT7, HT22, HT26

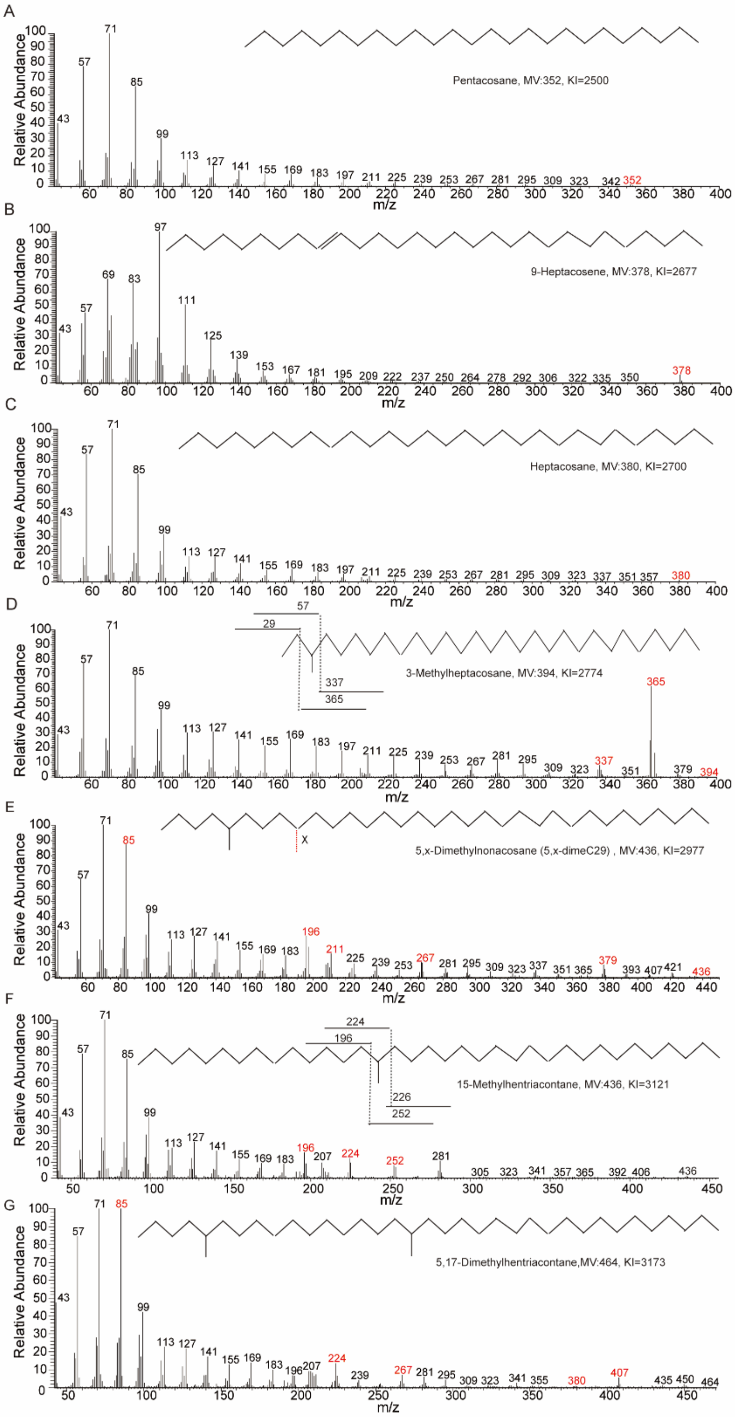

Figure S1. Mass spectra of main CHCs and their representative diagnostic ions and relative abundances. (A): pentacosane, (B): 9-heptacosene, (C): heptacosane, (D): 3-methyl-heptacosane, (E): 5,x-dimethly-nonacosane, (F): 15-methyl-hentriacontane, (G): 5,17-dimethyl-hentriacontane.

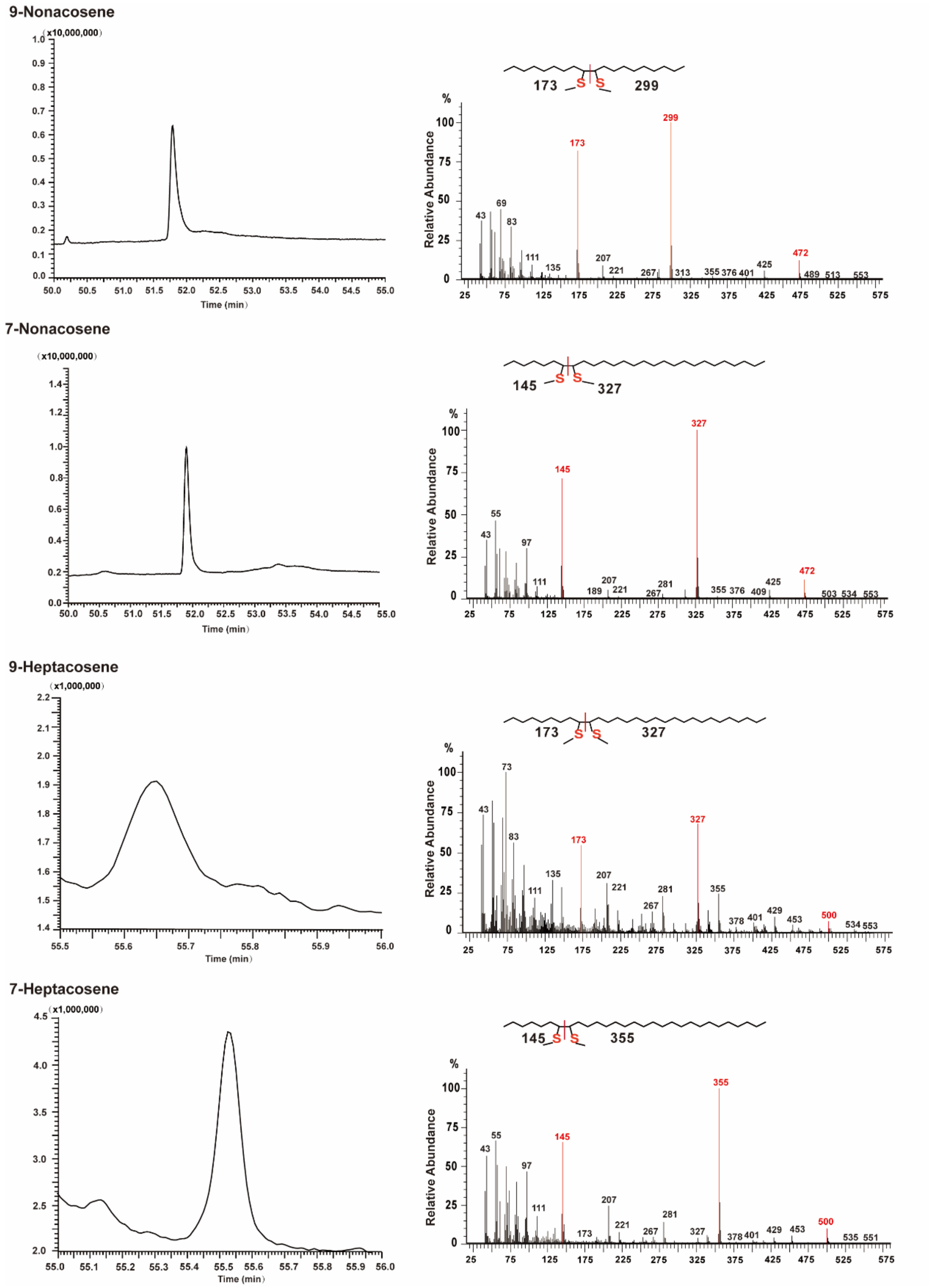

Figure S2. GC-MS showing the distribution of mono-DMDS adducts of standards.

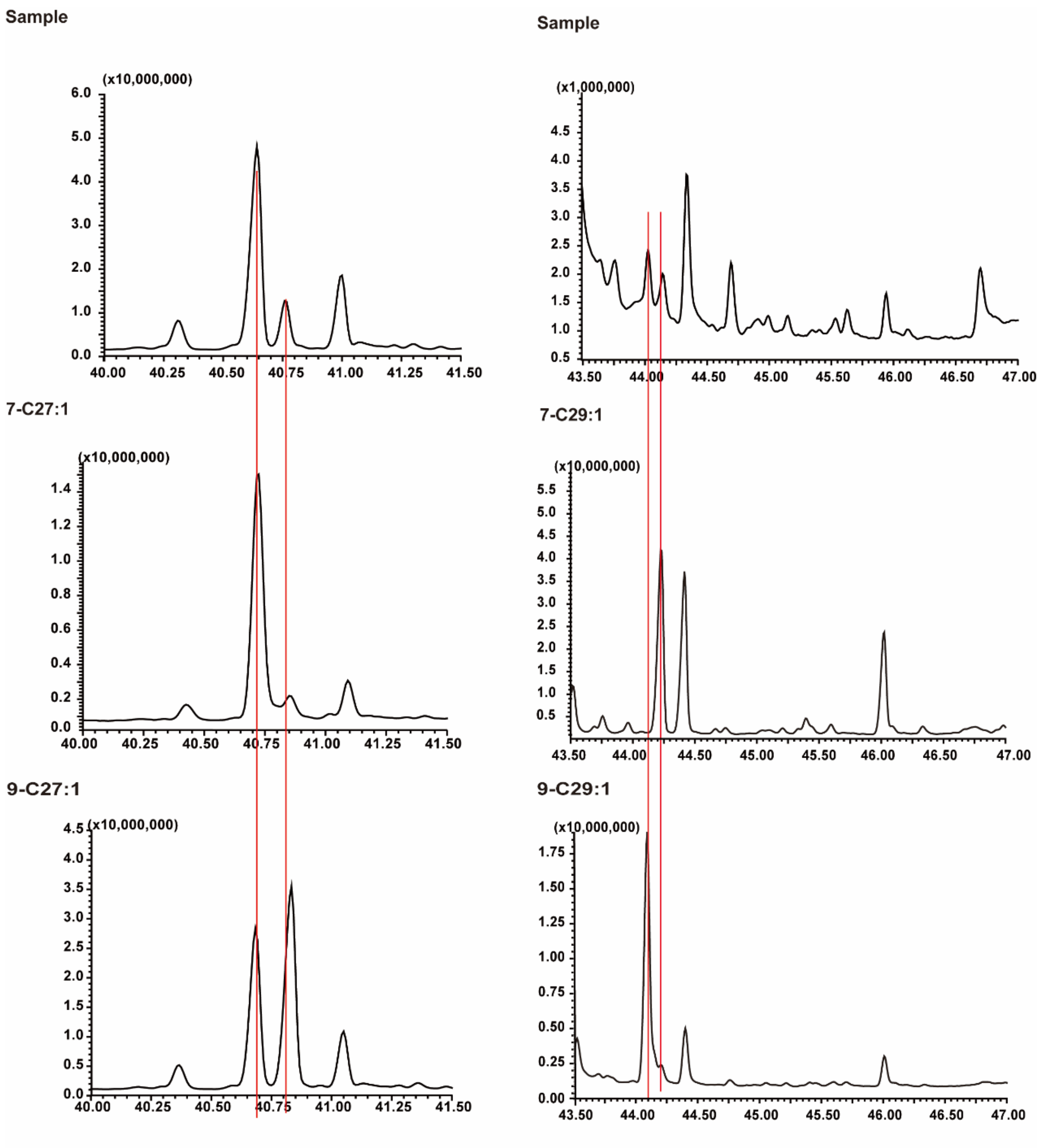

Figure S3. Total ion chromatograms (TICs) of sample and samples with heptacosene and nonacosene standards.
